# Effect of physical therapy for chronic obstructive pulmonary disease: A protocol for an updated systematic review of randomized controlled trial

**DOI:** 10.1097/MD.0000000000017241

**Published:** 2019-09-20

**Authors:** Hui Gao, Yuan Gao, Ping Sun, Jie Shen, Hui-juan Yao, Shi-dong Fu, Cheng Meng

**Affiliations:** Department of Respiratory Medicine, Yan’an People's Hospital, Yan’an, China.

**Keywords:** chronic obstructive pulmonary, effectiveness, physical therapy, safety

## Abstract

**Background:**

Previous studies have reported that physical therapy (PT) can be used for the treatment of chronic obstructive pulmonary disease (COPD). However, its effectiveness is still inconclusive. This systematic review will aim to assess its effectiveness and safety for the treatment of patients with COPD.

**Methods:**

All randomized controlled trials (RCTs) literatures of PT for COPD will be searched from the databases of Cochrane Central Register of Controlled Trials (CENTRAL), EMBASE, MEDILINE, Web of Science, Cumulative Index to Nursing and Allied Health Literature, Allied and Complementary Medicine Database, Chinese Biomedical Literature Database, China National Knowledge Infrastructure, VIP Information, and Wanfang Data from inception to the present without any language restrictions. Two reviewers will independently perform the study selection, data extraction, and methodological quality assessment. A third reviewer will be invited to resolve any disagreements occurred between 2 reviewers.

**Results:**

The primary outcome is lung function. The secondary outcomes include symptoms, health-related quality of life, mortality, and adverse events. The outcome data will be pooled by using the models of random-effects or fixed-effects according to the detected heterogeneity.

**Conclusion:**

The findings of this study will provide up-todated summary evidence for assessing the effectiveness and safety of PT for COPD.

## 1 Introduction

Chronic obstructive pulmonary disease (COPD) is a major public heath disorder around the world.^[1,2]^ It is manifested by dyspnea, chronic cough, production of sputum, and decreased physical activity.^[3,4]^ Many risk factors are reported to account for COPD, including tobacco smoke, passive smoking, air pollution, and so on.^[5–8]^ It has been reported that this disorder will become the third leading cause of death by 2030.^[9,10]^ The huge cost of COPD treatment imposes a very heavy burden for both families and society.^[11,12]^

Despite its high incidence, the managements of COPD still suffered from limited efficacy in many patients.^[4,13,14]^ Physical therapy (PT) has reported to benefits patients with COPD for reducing dyspnea, and improving respiratory muscle strength, lung function, and quality of life.^[15–20]^ Presently, although several reviews have addressed this issue,^[21–23]^ none of them have further assessed the effectiveness and safety of PT for COPD after more new randomized controlled trials have been published.^[15–18]^ Therefore, in this study, we will provide latest and updated evidence of systematic review to evaluate the effectiveness and safety of PT for COPD.

## 2 Methods and analysis

### 2.1 Objective

This study aims to evaluate the effectiveness and safety of PT for the treatment of patients with COPD.

### 2.2 Study registration

The protocol of this systematic review has been registered (PROSPERO CRD42019120813). We have prepared and designed this protocol according to the Preferred Reporting Items for Systematic Reviews and Meta-Analysis Protocol statement guidelines.^[24]^

### 2.3 Inclusion criteria for study selection

#### 2.3.1 Type of studies

Only randomized controlled trials (RCTs) of PT for COPD will be included in this systematic review. Any other studies such as Non-clinical trials, case reports, case series, Non-RCTs, and quasi-RCTs will all be excluded.

#### 2.3.2 Type of participants

Patients of any age with COPD, regarding race and sex will be considered. However, patients with lung cancer, and any other cancers transferred to the lung will be excluded.

#### 2.3.3 Type of interventions

Intervention of any types of PT alone will be included. However, the combination of PT with other treatments will be excluded. Control intervention will be the any kinds of therapies, such as medication, placebo, or any others except the any types of PT.

#### 2.3.4 Type of outcome measurements

Primary outcome is lung function. It can be measured by any instruments, such as forced expiratory volume in 1 second, forced vital capacity (FVC), forced expiratory volume in 6 seconds, peak expiratory flow, forced expiratory flow at 25% to 75% of FVC, and forced expiratory flow at 75% of FVC.

The secondary outcomes will include symptoms, such as dyspnoea, cough; health-related quality of life, as measured by any instruments, such as the 36-Item Short Form Health Survey; mortality; as well as the adverse events.

### 2.4 Search methods for the identification of studies

#### 2.4.1 Electronic searches

The databases of Cochrane Central Register of Controlled Trials (CENTRAL), EMBASE, MEDILINE, Web of Science, Cumulative Index to Nursing and Allied Health Literature, Allied and Complementary Medicine Database, Chinese Biomedical Literature Database, China National Knowledge Infrastructure, VIP Information, and Wanfang Data will be searched for the related trials of PT for COPD from inception to the present without any language restrictions. The detailed search strategy of CENTRAL is showed in Table 1. Similar strategies will be applied to other databases.

**Table 1:**
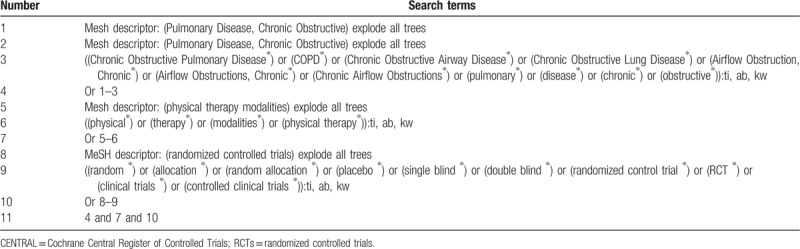
Search strategy applied in CENTRAL database.

#### 2.4.2 Search for other resources

The sources of clinical registration and reference lists of relevant studies will also be searched to identify any other eligible studies.

### 2.5 Data collection and analysis

#### 2.5.1 Study selection

**Figure 1:**
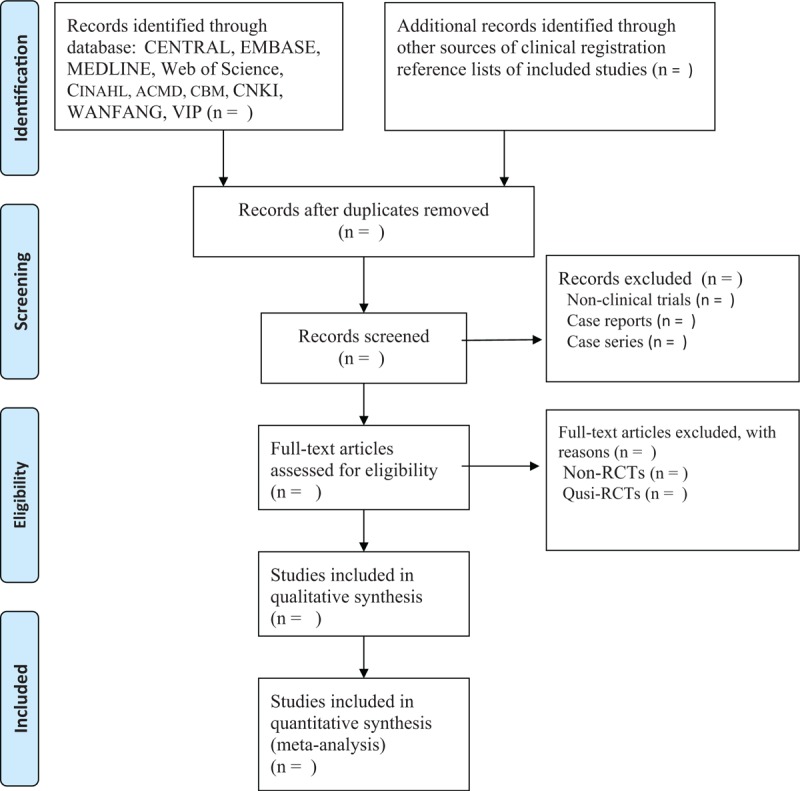
Process of study selection.

Two reviewers will independently conduct the study selection by scanning the titles and abstract summary initially, and then by reading the full texts according to the predefined inclusion criteria and exclusion criteria. Any divergences between 2 reviewers will be solved by discussion with a third reviewer. The study selection procedure is presented in Fig. 1.

#### 2.5.2 Data collection and management

Two reviewers will independently extract data from each included study according to the predefined standard data extraction form. The details of general information, such as first author, published year, region; design methods; interventions; outcome reports, and any other information are all extracted. Any differences of data collection between 2 reviewers will be resolved by a third reviewer through discussion.

#### 2.5.3 Risk of bias assessment

The methodological quality of all included trials will be assessed by using Cochrane risk of bias tool. It consists of 7 domains, and each domain is categorized as high risk of bias, unclear risk of bias, and low risk of bias. Two reviewers will independently evaluate the risk of bias, and disagreements will be resolved by discussion with a third author.

#### 2.5.4 Measurement of treatment effect

As for enumeration data, we will use risk ratio with 95% confidence intervals (CIs) to present it. As for continuous data, we will use mean difference or standardized mean difference with 95% CIs to present it.

#### 2.5.5 Dealing with missing data

If essential information and data are insufficient or missing, we will contact the original authors to request it. If we cannot get reply, then we will pool and analyze the current available data.

#### 2.5.6 Assessment of heterogeneity

The heterogeneity of pooled data will be identified by using the tests of *I*^2^ and *χ*^2^. If *I*^2^ is <50%, the heterogeneity is acceptable. Otherwise, the heterogeneity is considered as significant.

#### 2.5.7 Data synthesis

If the heterogeneity is acceptable, data will be pooled by using a fixed-effects model, and meta-analysis will be carried out using RevMan 5.3 software (Cochrane Community, London, UK). Otherwise, a random-effects model will be used; subgroup analysis and sensitivity analysis will be conducted to analyze potential factors that may result in high heterogeneity. If the heterogeneity remains substantial after subgroup analysis, a narrative summary will be performed.

#### 2.5.8 Subgroup analysis

If heterogeneity is significant, then subgroup analysis will be carried out in accordance with the different interventions, controls, and outcome measurement tools.

#### 2.5.9 Sensitivity analysis

Where appropriate, we will also conduct sensitivity analysis to eliminate the impact of low quality studies, as well as to assess the robustness of the pooled results data according to the different methodological qualities, and statistical models.

#### 2.5.10 Publication biases

We will conduct funnel plot to detect publication bias if sufficient studies (normally >10 studies) are included.^[25]^ In addition, Egg regression will also performed quantitative analysis.^[26]^

## 3 Discussion

This study protocol of systematic review will be performed to evaluate the effectiveness and safety of PT for patients with COPD. Although 3 previous reviews have addressed this issue, the latest one has been published for >8 years,^[21–23]^ and 4 more high quality RCTs regarding the effectiveness and safety of PT for COPD have been published after that.^[15–18]^

This systematic review will provide the up-to-dated evidence on the effectiveness and safety of PT for COPD. It will provide helpful evidence for both clinical practice and future studies.

## Author contributions

**Conceptualization:** Hui Gao, Yuan Gao, Hui-juan Yao, Shi-dong Fu, Cheng Meng.

**Data curation:** Hui Gao, Yuan Gao, Ping Sun, Jie Shen, Shi-dong Fu, Cheng Meng.

**Formal analysis:** Yuan Gao, Ping Sun, Jie Shen, Hui-juan Yao, Shi-dong Fu, Cheng Meng.

**Funding acquisition:** Yuan Gao.

**Investigation:** Yuan Gao.

**Methodology:** Hui Gao, Ping Sun, Jie Shen, Hui-juan Yao, Shi-dong Fu, Cheng Meng.

**Project administration:** Yuan Gao.

**Resources:** Hui Gao, Ping Sun, Jie Shen, Hui-juan Yao, Shi-dong Fu, Cheng Meng.

**Software:** Hui Gao, Ping Sun, Jie Shen, Hui-juan Yao, Shi-dong Fu, Cheng Meng.

**Supervision:** Yuan Gao.

**Validation:** Hui Gao, Yuan Gao, Hui-juan Yao, Cheng Meng.

**Visualization:** Hui Gao, Yuan Gao, Ping Sun, Jie Shen, Shi-dong Fu, Cheng Meng.

**Writing – original draft:** Hui Gao, Yuan Gao, Ping Sun, Jie Shen, Hui-juan Yao, Shi-dong Fu, Cheng Meng.

**Writing – review & editing:** Hui Gao, Yuan Gao, Ping Sun, Jie Shen, Hui-juan Yao, Shi-dong Fu, Cheng Meng.
